# RNA-Seq Analysis Illuminates the Early Stages of *Plasmodium* Liver Infection

**DOI:** 10.1128/mBio.03234-19

**Published:** 2020-02-04

**Authors:** Maria Toro-Moreno, Kayla Sylvester, Tamanna Srivastava, Dora Posfai, Emily R. Derbyshire

**Affiliations:** a Department of Chemistry, Duke University, Durham, North Carolina, USA; b Department of Molecular Genetics and Microbiology, Duke University, Durham, North Carolina, USA; Stanford University

**Keywords:** *P. berghei*, *Plasmodium*, RNA sequencing, liver stage, malaria, transcription

## Abstract

The apicomplexan parasites *Plasmodium* spp. are the causative agents of malaria, a disease that poses a significant global health burden. *Plasmodium* spp. initiate infection of the human host by transforming and replicating within hepatocytes. This liver stage (LS) is poorly understood compared to other *Plasmodium* life stages, which has hindered our ability to target these parasites for disease prevention. We conducted an extensive transcriptome sequencing (RNA-Seq) analysis throughout the Plasmodium berghei LS, covering as early as 2 h postinfection (hpi) and extending to 48 hpi. Our data revealed that hundreds of genes are differentially expressed at 2 hpi and that multiple genes shown to be important for later infection are upregulated as early as 12 hpi. Using hierarchical clustering along with coexpression analysis, we identified clusters functionally enriched for important liver-stage processes such as interactions with the host cell and redox homeostasis. Furthermore, some of these clusters were highly correlated to the expression of ApiAP2 transcription factors, while showing enrichment of mostly uncharacterized DNA binding motifs. This finding indicates potential LS targets for these transcription factors, while also hinting at alternative uncharacterized DNA binding motifs and transcription factors during this stage. Our work presents a window into the previously undescribed transcriptome of *Plasmodium* upon host hepatocyte infection to enable a comprehensive view of the parasite’s LS. These findings also provide a blueprint for future studies that extend hypotheses concerning LS gene function in P. berghei to human-infective *Plasmodium* parasites.

## Abstract (precis)

The LS of *Plasmodium* infection is an asymptomatic yet necessary stage for producing blood-infective parasites, the causative agents of malaria. Blocking the liver stage of the life cycle can prevent clinical malaria, but relatively less is known about the parasite’s biology at this stage. Using the rodent model P. berghei, we investigated whole-transcriptome changes occurring as early as 2 hpi of hepatocytes. The transcriptional profiles of early time points (2, 4, 12, and 18 hpi) have not been accessible before due to the technical challenges associated with liver-stage infections. Our data now provide insights into these early parasite fluxes that may facilitate establishment of infection, transformation, and replication in the liver.

## INTRODUCTION

*Plasmodium* spp., the causative agents of malaria, are eukaryotic parasites with a largely conserved and complex life cycle that begins in the mammalian host by invasion of hepatocytes. In these host cells, a single parasite, termed a sporozoite, will transform and then replicate asexually to form thousands of merozoites, or blood-infective forms (1). After maturation and release from the liver, parasites replicate within erythrocytes, causing the clinical manifestation of malaria. Some parasites differentiate into sexual forms (gametocytes) that are ingested by an *Anopheles* mosquito during a blood meal. In the mosquito, female and male gametocytes undergo sexual reproduction, and a series of developmental changes lead to a transformation into sporozoites. Inoculation of these sporozoites in the host via a mosquito bite perpetuates the life cycle (2). Despite the significant global burden of malaria (3), our molecular understanding of the *Plasmodium* life cycle is incomplete, hindering our ability to target these parasites to prevent disease and reduce transmission. In particular, the changes that enable sporozoites to transform and then develop within hepatocytes are largely unknown.

Transcriptomic studies have been instrumental in revealing gene expression variation that accompanies stage transitions and developmental processes in *Plasmodium*. Subsequent analyses of these data have also identified transcription factors (TFs) that are critical for controlling parasite progression at various stages [reviewed in reference 4]). However, only a few transcriptome analyses have been completed in the liver stage (LS) relative to other parasite forms, likely owing to the technical challenges associated with studying this stage. Still, these studies have provided important insight into LS-specific biological processes (5), including hypnozoite markers (6, 7), and comparative gene expression analysis with other stages (8), even at a single-cell resolution (9). These studies examined gene expression upon the establishment of a LS-trophozoite (24 h postinfection [hpi] and thereafter); however, the early stages of LS infection (0 to 24 hpi) for any *Plasmodium* species remain unresolved.

Our current understanding of the early stages of LS development comes from ultrastructural (10) and immunofluorescence (11) studies. Upon traversal and invasion of hepatocytes, rod-shaped sporozoites expulse unnecessary organelles into the parasitophorous vacuole (PV), which is accompanied by the formation of a protrusion, a bulbous expansion, and a transformation into a spherical, replication-competent trophozoite (10). Although this metamorphosis is obvious at the cellular level, the molecular events underpinning this sequence of events remain obscure. Previous studies have examined the gene expression of sporozoites grown axenically since sporozoites can complete this transformation extracellularly if activated by bovine serum albumin, calcium, and a temperature shift (12, 13). Yet, axenically grown sporozoites show reduced viability and poor developmental capacity compared to intracellular parasites, suggesting an important role of host pathways in this process. Indeed, a recent study showed that activation of the host GPCR CXCR4 is necessary for proper parasite metamorphosis (11), highlighting the need to study parasite transformation, and all its subsequent development, in the context of the host cell.

Here, we present a transcriptomic survey of the early and middle LS of Plasmodium berghei infecting human hepatoma cells. The rodent P. berghei and P. yoelii LS models are routinely used to study this stage due to their genetic accessibility and tractability relative to human-infective counterparts. Our data set includes seven time points, from 2 to 48 hpi, making it the most comprehensive transcriptomic analysis of the *Plasmodium* LS to date. We describe changes in gene expression associated with the early stages of *Plasmodium* intracellular development in the LS and show that upregulation of most genes important for exoerythrocytic form maturation occurs as early as 12 hpi. This finding suggests genes important for late-LS development are subject to dynamic expression or translational repression until protein expression is necessary. Furthermore, using coexpression analysis we identified functionally enriched gene clusters with distinct expression patterns and discovered dozens of potential regulatory DNA motifs associated with these genes. Overall, our work completes the life cycle of this important model organism, P. berghei, from the transcriptomic perspective, providing a resource for exploring stage-specific expression of genes and thus advancing our understanding of *Plasmodium* biology.

## RESULTS

### RNA-Seq of early- and mid-*P. berghei* liver stages.

**FIG 1:**
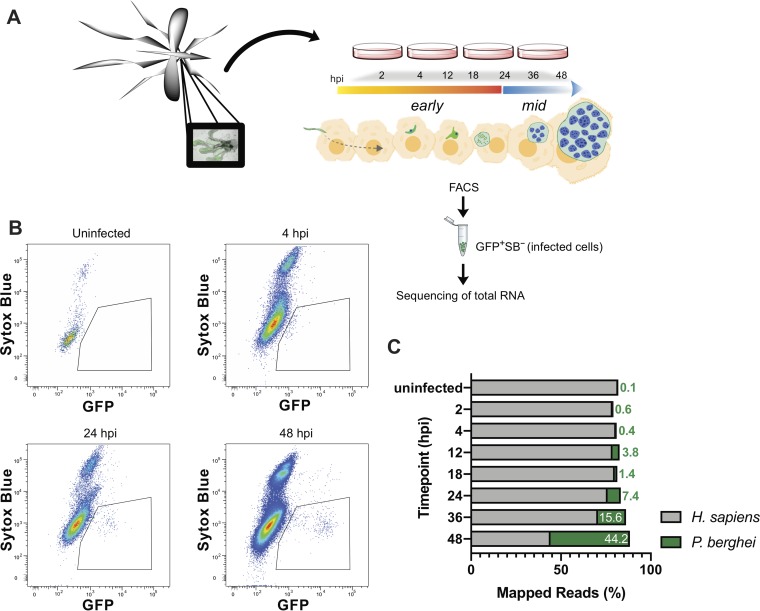
Experimental design for RNA-Seq of early and mid-stages of P. berghei liver infection. (A) Experimental design schematic. Female *Anopheles* mosquitoes were dissected and GFP-expressing P. berghei sporozoites were harvested to infect HuH7 or HepG2 cells. Cells were harvested 2, 4, 12, 18, 24, 36, or 48 hpi and FACS sorted to enrich viable P. berghei-infected cells for RNA collection. (B) Representative flow cytometry fluorescence dot plots indicating the population of GFP^+^ Sytox Blue^–^ cells that were collected at various time points. (C) Relative percentage of transcripts mapping to P. berghei or H. sapiens at various times postinfection. Uninfected samples correspond to naive uninfected cells treated with debris from dissected male *Anopheles* mosquito salivary glands. The data are medians of two to five biological replicates.

During the course of the LS, sporozoites undergo morphological changes and rapid replication. To investigate differentially expressed transcripts that flux during this stage, HuH7 or HepG2 hepatoma cells were infected with green fluorescent protein (GFP)-expressing P. berghei ANKA sporozoites. At various times postinfection, samples were harvested, and 1,000 to 3,000 P. berghei-infected cells were collected by fluorescence-activated cell sorting (FACS) (Fig. 1A). A poor understanding exists for the early- and mid-LS; therefore, greater sampling was acquired before 24 hpi at 2, 4, 12, and 18 hpi (early). Previously analyzed mid-LS samples at 24 and 48 hpi were collected to enable comparison to other studies, as well as 36 hpi, which has not been previously evaluated. *Plasmodium* infection in liver cells is highly heterogeneous, with ∼50% of sporozoites that invade liver cells failing to establish productive infections (14, 15). We ensured selection of populations enriched for productive infections within viable host cells by isolating cells that are both infected and have an uncompromised membrane (GFP^+^ Sytox Blue^–^). FACS analysis indicates that the population of infected cells (GFP^+^) shifts as a function of time, consistent with proper intrahepatic parasite maturation (Fig. 1B). Further, our gating excluded nonviable host cells (Sytox Blue^+^). In our method, we sorted directly into lysis buffer. RNA was then extracted in each sample using a Clontech kit for ultralow input RNA. Samples were evaluated for concentration and quality using a Qubit and Bioanalyzer, respectively, and analyzed by RNA-Seq if they met quality controls. To facilitate robust analysis, sample collection continued until a minimum of three replicates per time point was acquired, which yielded a final range of three to eight replicates.

All samples were aligned to H. sapiens and P. berghei for analysis. Since parasite nuclear division does not occur until mid-LS, <4% of the reads mapped to P. berghei before 24 hpi. This percentage rises continuously during mid-LS, when the parasite undergoes nuclear division, and by 48 hpi ∼45% of the reads correspond to P. berghei (Fig. 1C). Here, we are focused on parasite processes that control development within hepatocytes; thus, principal-component analysis (PCA) was completed on P. berghei data after the removal of batch effects. PCA revealed no major differences between the parasite transcriptomes obtained by infecting HepG2 or Huh7 cells (see Fig. S1A in the supplemental material), but a general clustering of replicates by genotype (time point) was observed (Fig. S1B). Of note, PCA showed strong separation at 4 and 2 hpi, with the latter grouping well with sporozoites, highlighting the parasite transformations that must occur during these 2 h.

10.1128/mBio.03234-19.1 FIG S1 (A) PCA of all samples after removal of batch effects. Colors correspond to genotypes/timepoints (spz, as well as 2, 4, 12, 24, 36, and 48 hpi). Shapes designate the cell line used for the infection. (B) Hierarchical clustering of the different samples based on all genes using a correlation distance with complete linkage. The sample names are colored by genotype (legend in left panel). The PCA showcases major sources of variation in the data, while the hierarchical clustering plot specifically identifies the most similar samples (based on correlation of all genes) and groups them together in a step-wise fashion. For the PCA analysis, we showcase the first two components to illustrate that while the largest source of variation does not separate out the time points (genotypes); it shows that cell type and sequencing batch are not major contributors to the variation of the data. Nonetheless, the hierarchical clustering shows that, overall, our time points tend to cluster together, based on correlation of all genes. Download FIG S1, TIF file, 0.3 MB. Copyright © 2020 Toro-Moreno et al. 2020 Toro-Moreno et al. This content is distributed under the terms of the Creative Commons Attribution 4.0 International license.

**FIG 2:**
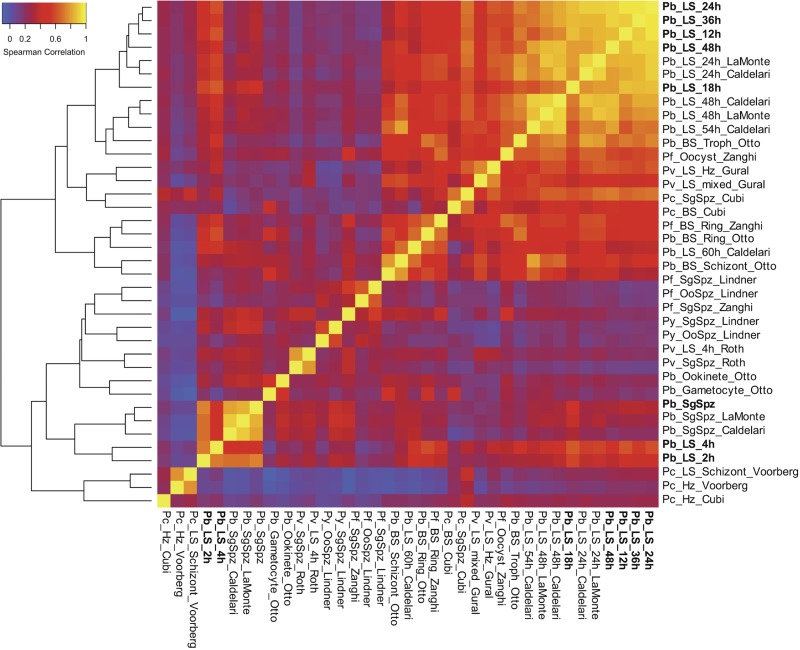
Overview of *Plasmodium* transcriptome analyses. Hierarchical clustering of gene expression data sets from different stages of the *Plasmodium* life cycle (7, 8, 25, 31, 33, 51–53). Data sets generated in this study are in bold. Clustering is based on Spearman correlation coefficients calculated and plotted using R. Refer to Table S1 in the supplemental material for information regarding the data sets used to generate this figure.

To analyze our data set in the context of the entire *Plasmodium* life cycle, we calculated Spearman correlations on our data, as well as previously published *Plasmodium* transcriptomic data from sporozoites, the asexual blood stage (ABS), gametocytes, ookinetes, hypnozoites, and the LS (see Table S1 in the supplemental material). This analysis spanned data obtained from P. berghei, P. yoelii, P. cynomolgi, P. vivax, and P. falciparum. Consistent with previous reports, the LS was more similar to the ABS than to gametocytes and ookinetes (8). Indeed, we observe two general groups comprising of (i) mostly metabolically active, intracellular stages (LS and ABS) and (ii) mostly motile, extracellular stages (Fig. 2). Notably, early liver stages of P. berghei and (axenic) P. vivax (LS_2h/4h) fell into the latter group, being more highly correlated to sporozoites, ookinetes, and gametocytes than to other LS time points.

10.1128/mBio.03234-19.6 TABLE S1 Datasets used in Spearman correlation analysis (Fig. 2). Download Table S1, DOCX file, 0.02 MB. Copyright © 2020 Toro-Moreno et al. 2020 Toro-Moreno et al. This content is distributed under the terms of the Creative Commons Attribution 4.0 International license.

### Early liver-stage transcriptome of *P. berghei*.

**FIG 3:**
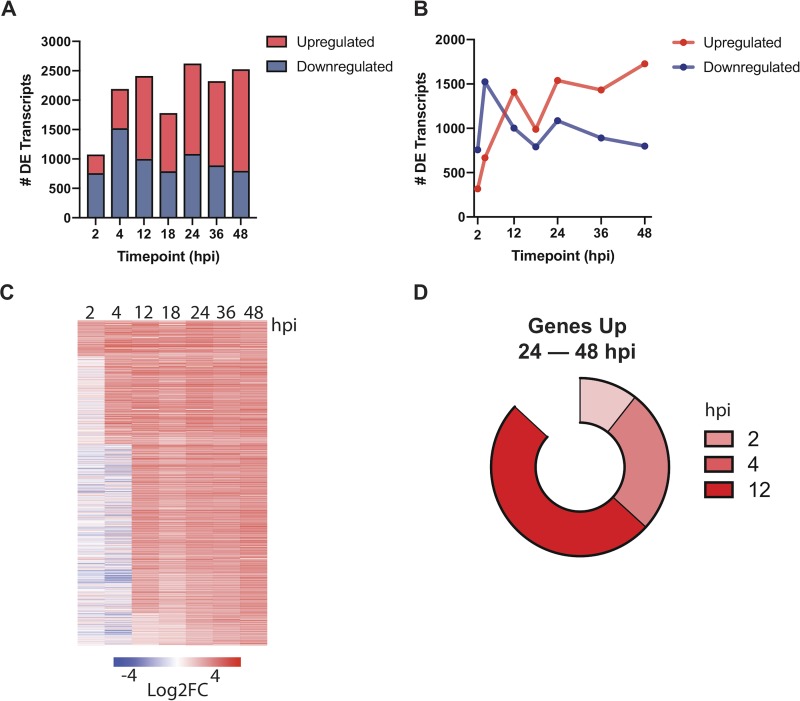
Dynamic gene regulation throughout liver-stage P. berghei development. (A and B) Total (A) and upregulated (red)/downregulated (blue) (B) differentially expressed (DE) transcripts (*q *< 0.01) are shown at each time point. (C) Expression profiles of 1,197 genes upregulated at 24, 36, and 48 hpi ordered based on the time point they were first observed to be upregulated. Expression is shown as the log_2_-fold change (Log2FC) versus sporozoite samples. (D) The proportions of genes upregulated throughout late-stage development (24, 36, and 48 hpi) are divided by when they were first observed to be upregulated (2, 4, or 12 hpi).

Thousands of statistically significant differentially expressed transcripts were detected at early-LS time points, with most of these transcripts being downregulated at 2 and 4 hpi and then upregulated at 12 hpi with respect to sporozoites (Fig. 3A and B; Data Set S1). This shift suggests a change from gene suppression to activation as the parasite exits the early stage of intrahepatic development. As expected, genes important for host cell traversal and invasion, such as *CELTOS*, *SUB2*, and *CSP*, were downregulated at 2 hpi, concurrent with the upregulation of genes important for nutrient acquisition (*ZIP1*, *TPT*, and *NT1*), reflecting the establishment of the infection in the host cell. Unsurprisingly, at these early stages, we also observed strong upregulation of *EXP2* and *PV2*, which encode parasitophorous vacuole membrane (PVM)-associated proteins, together with several predicted exported proteins of unknown function, indicating that early (<4 hpi) establishment and remodeling of the PVM is essential for parasite LS maturation. Interestingly, we observed that *LYTB* (IspH), the last enzyme in the isoprenoid biosynthesis pathway in the apicoplast, is among the most-upregulated genes at both 2 and 4 hpi (Table S2). Apicoplast pathways are important potential drug targets for the development of LS antimalarials but are not known to be involved in early-LS processes.

10.1128/mBio.03234-19.7 TABLE S2 Top most upregulated genes at 4 hpi. Download Table S2, DOCX file, 0.01 MB. Copyright © 2020 Toro-Moreno et al. 2020 Toro-Moreno et al. This content is distributed under the terms of the Creative Commons Attribution 4.0 International license.

10.1128/mBio.03234-19.9 DATA SET S1 Differential expression. Gene expression (RNA-Seq) of P. berghei for all the datasets analyzed in this study. The fold change was determined for each time point versus the sporozoite samples. Columns are as follows: GeneID, GeneName, gene product description (PlasmoDB); IsCoding, “is the gene a known protein-coding gene?”; LogFC, log_2_(fold change); lfcSE, standard error of the log_2_(fold change); stat, Wald test statistic; pvalue; padj, FDR-corrected *P* value; <SampleID>, Normalized expression value for specific <SampleID>. Download Data Set S1, XLSX file, 6.1 MB. Copyright © 2020 Toro-Moreno et al. 2020 Toro-Moreno et al. This content is distributed under the terms of the Creative Commons Attribution 4.0 International license.

Translational regulation of *Plasmodium* transcripts has been extensively documented, and it is known to play a pivotal role during developmental transitions in the life cycle. We found pervasive upregulation of most of the functionally characterized translational regulators in *Plasmodium*, at the exclusion of *PUF1* and *PUF2*, which appeared to be dramatically downregulated compared to their high expression in sporozoites*. DOZI*, *ALBA1*, *ALBA2*, and *ALBA4* were upregulated as early as 4 hpi (log_2_-fold change [Log2FC] < 2, *q *< 0.01) (Fig. S2). Moreover, among the most differentially expressed transcripts at 2 and 4 hpi, there was an enrichment of genes involved in RNA-protein complexes and interactions, such as *SR1*, *NOP10*, *CBF5*, *RPS12*, and *NAPL* (Table S3). Thus, translational regulation likely plays an important role in the early stages of *Plasmodium* infection of the liver.

10.1128/mBio.03234-19.2 FIG S2 Expression profile of P. berghei translational regulators in the liver stage. The gene IDs and names are shown in legend. Expression is shown as the log_2_(fold change). Download FIG S2, TIF file, 1.1 MB. Copyright © 2020 Toro-Moreno et al. 2020 Toro-Moreno et al. This content is distributed under the terms of the Creative Commons Attribution 4.0 International license.

10.1128/mBio.03234-19.8 TABLE S3 Top most upregulated genes at 12 hpi. Download Table S3, DOCX file, 0.01 MB. Copyright © 2020 Toro-Moreno et al. 2020 Toro-Moreno et al. This content is distributed under the terms of the Creative Commons Attribution 4.0 International license.

At ∼24 hpi and thereafter, the single-nucleated trophozoites replicate and subsequently mature into LS schizonts, each harboring tens of thousands of nuclei. Previous work examining the LS transcriptome at these middle stages identified hundreds of differentially expressed genes involved in translation, metabolism, protein trafficking, and redox processes (5, 8). Since we saw a strong correlation between 12 hpi and mid-LS (Spearman correlation = 0.837 to 0.949; Fig. 2), we sought to determine how early a statistically significant upregulation of the core mid-LS transcriptome could be observed in our data set. We found 1,197 genes in our data set that are significantly upregulated at 24, 36, and 48 hpi compared to sporozoites (*q *< 0.01), constituting about 20% of the P. berghei genome (Fig. 3A and B). Interestingly, we found that 87% of transcripts that are upregulated throughout the mid-LS (24 hpi through 48 hpi) are upregulated as early as 12 hpi (Fig. 3C). More specifically, 50% of the genes that are upregulated in the mid-LS are first observed to be upregulated at 12 hpi (Fig. 3D).

### Coexpression analysis identifies functionally enriched gene clusters.

**FIG 4:**
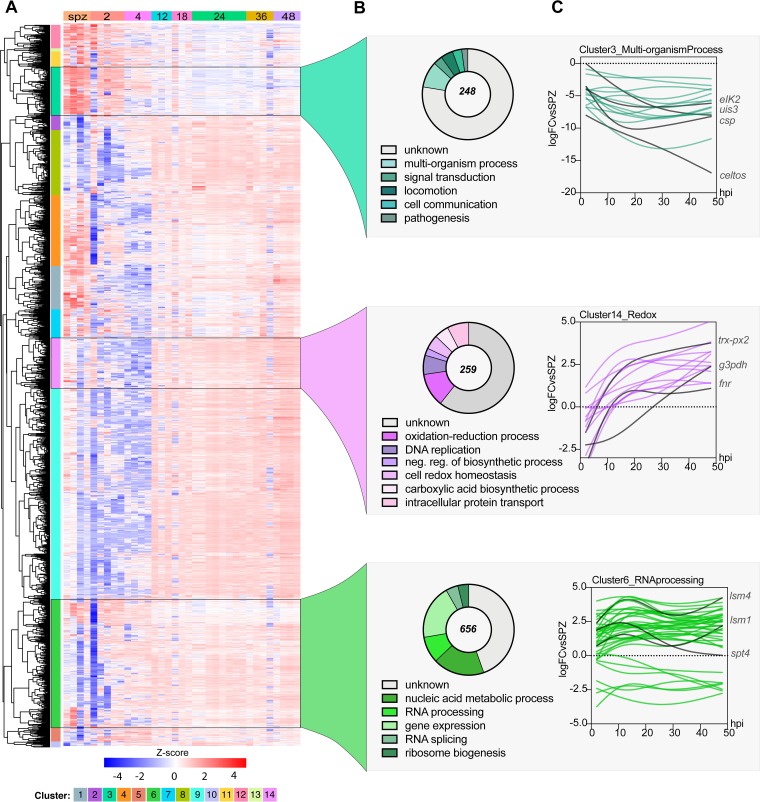
Coexpression analysis identifies enriched processes during P. berghei development in hepatocytes. (A) Hierarchical clustering using a correlation distance with complete linkage of all genes significant (FDR ≤ 5%) in at least one of the analyses. Gene expression is z-score transformed. (B) GO enrichment analysis (biological process) of enriched clusters 3, 14, and 6. Representative GO terms (*P* < 0.01) and their respective number of genes (pie chart) are shown. The total numbers of genes in each cluster are shown at the center of the pie chart. (C) Spline models of gene expression data for all the genes in the top-scoring GO term in each cluster. Key genes in each group and their expression patterns are highlighted in red. Refer to Data Set S2 in the supplemental material for complete GO analysis of all clusters.

To identify coexpression patterns that may inform future functional studies, we performed a clustering analysis of the k-means for all differentially expressed genes for all of the samples included in our data set. Fourteen clusters emerged from this hierarchical clustering analysis (Fig. 4A; see Data Set S2 in the supplemental material). These clusters could be further grouped within three major coexpression patterns when columns were grouped by sample genotype (time point). The first major cluster group (clusters 3, 11, and 13) includes genes that are upregulated early during infection (sporozoite [spz], 2 and 4 hpi) and are generally downregulated throughout the rest of LS infection, such as *ETRAMP*s and *SPELD*. The second major cluster group (clusters 1, 2, 4, 7, 8, 9, 12, and 14) includes genes that are downregulated during the early stages of infection but are then consistently upregulated from 24 to 48 hpi. The third major cluster group (clusters 5, 6, and 10) includes genes that are upregulated throughout the LS.

10.1128/mBio.03234-19.10 DATA SET S2 Cluster analysis. This file shows the genes that belong to each coexpression cluster and their characterization. Columns are as follows: GeneID; GeneName, gene product description (PlasmoDB); GO terms, GO terms associated with each cluster (*P* < 0.01); notable genes, selected genes from each cluster; DNA motif, enriched DNA motifs for each cluster discovered through the DREME (E value < 0.05). E value, the enrichment value for each DNA motif. Download Data Set S2, XLSX file, 0.1 MB. Copyright © 2020 Toro-Moreno et al. 2020 Toro-Moreno et al. This content is distributed under the terms of the Creative Commons Attribution 4.0 International license.

To investigate possible enrichment of biological processes of coexpressed genes, we analyzed each cluster by gene ontology (GO). Such analyses revealed the enrichment of various GO terms for each of the clusters (*P* < 0.01). We prioritized clusters for which at least one GO term was enriched by a *p-adj* (Bonferroni) value of <0.01. Cluster 3 stood out as highly enriched despite 142 of the total 248 genes in this cluster not being annotated. For this cluster, enrichment analysis indicated significant enrichment of “interspecies interaction” (GO:0044419, *P* < 1.91E–07), as well as locomotion (GO:0040011, *P* < 0.0005679) and signal transduction (GO:0007165, *P* < 0.00049572) (Fig. 4B). Genes in this cluster are highly expressed in sporozoites and thus appear to be strongly downregulated during infection (Fig. 4C). In agreement with this result, this cluster includes genes that have been previously shown to play an important role during invasion (*CELTOS*, *SPECT1*, and *TRAP*), interactions with the host liver cell (*UIS3*, *UIS4*, *CSP*, *p36*, and *p52*), and translational control of LS-specific transcripts (*UIS2*, *PUF1*, and *PUF2*) (16).

Cluster 14 was enriched for “oxidation-reduction process” (GO:0055114, *P* < 9.01E–05), “DNA replication” (GO:0006260, *P* < 0.00164223), and “intracellular protein transport” (GO:0006886, *P* < 0.00941436) (Fig. 4B). In this group, genes involved in redox-regulatory processes (*FNR* and *TRX-PX2*), as well as biosynthetic genes such as *G3PDH*, can be found. The expression of genes under the redox group appears to peak by ∼12 hpi and then remains stably upregulated throughout infection. This expression pattern highlights the need for this machinery to mitigate potential stress due to the dramatic parasite replication and growth that is initiated at ∼24 hpi (Fig. 4C). Little is known about redox biology in *Plasmodium* parasites, particularly during the LS, but these processes have historically been key pathways for drug discovery. Indeed, atovaquone, a drug for malaria prophylaxis in combination with proguanil, inhibits LS parasites *in vitro* by impairing mitochondrial redox metabolism by targeting the cytochrome *bc*_1_ complex (17). This data set may serve as a starting point to discover more LS targets involved in redox metabolism. Furthermore, although not enriched in our GO analysis, we observed that several important liver-specific genes are found in this cluster, such as *IBIS1*, *LISP1*, and *LISP2*. Finally, in cluster 6, we saw enrichment of core functions such as “gene expression” (GO:0010467, *P* < 5.71E–05) and “RNA processing” (GO:0006396, *P* < 5.98E–06), which contains 656 genes. As expected of housekeeping functions, these genes appear to be expressed throughout LS infection.

Our analysis identified several clusters with enriched GO terms, some of which accurately describe the known LS biology at different time points. Although GO enrichment provided a useful assessment of differentially expressed processes, we note that it is limited in its reach in *Plasmodium* compared to other model organisms since ∼40% of the genome remains unannotated. Hence, to further explore the composition of these coexpression clusters, we made use of the Rodent Malaria genetically modified Parasite Database (RMgmDB) to provide phenotypic information about our clusters throughout the life cycle (18). Interestingly, we observed that while most clusters have a high proportion of genes for which disruption resulted in phenotypes across the entire life cycle, only a few clusters had genes that displayed phenotypes exclusively in sporozoite and/or liver stage (Fig. S3). Specifically, clusters 3 and 14 had the highest percentage of spz/LS-specific genes (13 and 9%, respectively), reinforcing the potential for identifying new LS drug and vaccine targets within these clusters.

10.1128/mBio.03234-19.3 FIG S3 Phenotypic overview of coexpression clusters. (A) Percent of genes in each cluster that are reported to have been “successfully disrupted” in the RMgmDB. (B) Number of genes in each cluster for which successful disruption resulted in distinct phenotypes from WT parasites. “Spz/LS” (pink) denotes mutants displaying phenotypes only in sporozoites or liver stages; “all life cycle” (teal) display phenotypes across all life cycle (asexual blood stage, gametocyte/gamete, fertilization and ookinete, oocyst, sporozoite, and liver stage); “other” (grey) display phenotypes distinct from the two above. All of the data are from the RMgmDB (18). Download FIG S3, TIF file, 1.2 MB. Copyright © 2020 Toro-Moreno et al. 2020 Toro-Moreno et al. This content is distributed under the terms of the Creative Commons Attribution 4.0 International license.

### Expression dynamics of AP2 transcription factors.

**FIG 5:**
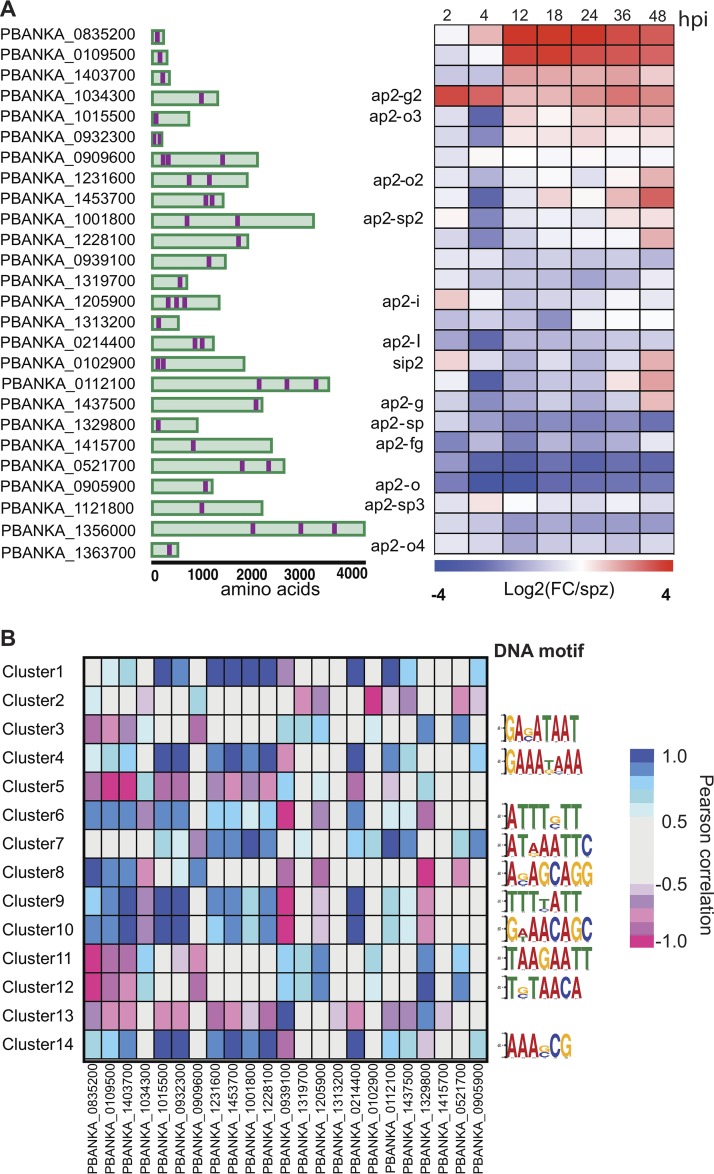
Expression of P. berghei AP2 transcription factors in the liver stage. (A) Gene IDs of the 26 AP2 transcription factors in the P. berghei genome, their respective protein architecture schematic (with AP2 displayed in purple), and their corresponding expression as the log_2_-fold change versus spz at each time point in the LS. (B) Heat map of Pearson correlations between AP2 transcription factors and the average expression of all genes in each cluster (left). The top most enriched DNA motif for each cluster discovered through the DREME pipeline is shown (right). Refer to Data Set S2 for a complete set of motifs and their respective enrichment scores.

Transcriptional regulation of gene expression has been extensively studied in the intraerythrocytic developmental cycle (IDC) and mosquito stages of P. berghei and P. falciparum. The AP2 transcription factors (TFs), comprised of 26 genes in P. berghei, are the best-characterized family of TFs in apicomplexans (Fig. 5A). AP2s are known to regulate *Plasmodium* transitions into different developmental stages and have emerged as key factors leading to both sexual commitment and sex differentiation (reviewed in reference 4). Unsurprisingly, we observed that AP2 genes with established functions in mosquito stages (*AP2-O* and *AP2-O2*) and those involved in sporozoite development (*AP2-SPs*) are downregulated throughout the liver stages (Fig. 5A). The only ApiAP2 TF known to play a role in LS development is *AP2-L*. *AP2-L*^–/–^ parasites are able to traverse and invade liver cells but arrest in the schizont stage (19). *AP2-L* transcripts are abundant in sporozoites and thus appear to be strongly downregulated during infection, as early as 2 hpi (Fig. 5A).

We observe strong upregulation (∼3-fold) of *AP2-G2* at 2 and 4 hpi. AP2-G2 has been shown to act as a repressor during the blood stage (BS) and gametocyte development and to have different targets during these stages (20, 21). A group of such targets corresponds to the liver-specific genes *LISP1* and *TREP*, which are important for LS schizont maturation and are expressed during late-LS infection (21). Interestingly, we observe that *AP2-G2* expression is negatively correlated to the average expression of the main clusters harboring this set of genes, including clusters 1, 9, and 14 (see Data Set S2 in the supplemental material). Thus, it is plausible that during the first hours of infection, *AP2-G2* acts as a repressor of genes involved in later stages of LS development, many of which remain uncharacterized.

Interestingly, we observed significant upregulation of the uncharacterized ApiAP2s PBANKA_0835200 and PBANKA_0109500 throughout the LS starting at 12 hpi, in contrast to the early upregulation of *AP2-G2*. Although still functionally uncharacterized, their orthologs in P. falciparum have been recently shown to coexpress during differentiation in gametocytogenesis and to be inversely correlated to genes involved in ABS development. This expression pattern suggests they may have a role as corepressors of genes involved in the ABS (22). In our data set, we observe a strong correlation with clusters 11 and 12 (both negative) and with cluster 8 (positive).

We sought to identify enriched DNA motifs in each of the coexpression clusters by analyzing the 5′ untranslated region sequences (1 kb) of their genes against the upstream sequence for all of the genes in other clusters using DREME (Data Set S3) (23). While genes in clusters 1, 2, 5, and 13 lacked any enriched DNA motifs, *de novo* discovery uncovered hundreds of DNA motifs in the remaining clusters, with the topmost enriched motif shown in Fig. 5B. We found that the most significant motif in cluster 12 (T[G/C]TAACA) matched the motif recognized by ApiAP2 PBANKA_0521700 (GTGTTACAC, *P* < 1.28E–05). This cluster included genes that are mostly downregulated throughout the LS until the later time points in our time series, such as the BS schizont-specific genes *SERA2* and *SERA3*. In addition, PBANKA_0521700 expression was strongly correlated to cluster 12 (*r* = 0.83, *P* < 0.021), suggesting this cluster might harbor previously unknown targets of this ApiAP2 (Fig. 5B).

## DISCUSSION

Our data provide novel insights into gene expression fluxes throughout *Plasmodium* development within hepatocytes. The transcriptional blueprints provided by our time series enables comparison of early-, mid-, and late-LS parasite processes for the first time. We found 146 genes exclusively upregulated early, such as *EIF5*, and 482 genes, including *SERA1* and *LISP2*, exclusively upregulated in the mid-LS (Fig. S4). Furthermore, our data sets recapitulated well-established gene expression patterns of key LS genes and overall were largely in agreement with recently reported data sets, supporting the validity of our approach. Through our analysis, we identified a key shift in parasite gene expression that occurs at 12 hpi and the role of transcription factors in driving LS maturation. Specifically, we explored potential transcriptional regulation of coexpressing genes by analyzing their upstream sequences for enrichment of potential DNA binding motifs and their correlation to P. berghei AP2 transcription factors. Our results revealed an association between the uncharacterized PBANKA_0521700 AP2 TF and cluster 12. PBANKA_0521700 is preferentially expressed in the ring stages of the IDC and is refractory to disruption in the BS (21, 24), hampering functional studies of this gene. Our data, in conjunction with previously reported P. berghei RNA-Seq (8, 21, 24, 25) and single-cell studies covering the entire life cycle (9), could be useful to refine hypotheses about the functions and targets of this TF, as well as other AP2 TFs.

10.1128/mBio.03234-19.4 FIG S4 Venn diagram illustrating the overlap of the upregulated (A) and downregulated (B) genes at early (2, 4, 12, or 18 hpi) and mid-stages (24, 36, or 48 hpi). Download FIG S4, TIF file, 0.1 MB. Copyright © 2020 Toro-Moreno et al. 2020 Toro-Moreno et al. This content is distributed under the terms of the Creative Commons Attribution 4.0 International license.

Although AP2 TFs have been at the center of gene expression studies in *Plasmodium*, novel “omics” approaches have begun uncovering other layers of gene regulation. Indeed, posttranscriptional regulation, such as *N*^6^-methyladenosine (m^6^A) of mRNA and alternative splicing, have recently been recognized as essential for fine-tuning gene expression in blood and sexual stages (26, 27). In particular, disruption of the splicing factor *Pb*SR-MG was shown to perturb sex-specific alternative splicing, thus demonstrating its role as a cellular differentiation regulator (28). Interestingly, we observed a dramatic upregulation of the splicing factor SR1 coinciding with the parasite’s metamorphoses in the LS, hinting at an important role for alternative splicing during this stage. Future reverse genetic studies may help establish a role for alternative splicing in the LS.

A well-documented form of gene expression regulation in *Plasmodium* occurs at the translational level. Translational repression (TR) of hundreds of transcripts has been reported at most stages of the P. berghei life cycle (29). TR is particularly pervasive in the sporozoite transition from the mosquito to the mammalian host (30, 31). During this transition, hundreds of transcripts that are highly expressed in sporozoites are stored in mRNA granules, until infection of the host relieves this repression, resulting in protein translation. The extent to which a TR program operates in the LS is currently unknown. However, we observed that ∼50% of all transcripts upregulated after 24 hpi are also upregulated at 12 hpi, including some with known roles in LS schizont maturation (*IBIS1* and *BP2*). Furthermore, we observed the upregulation of several known translational regulators, e.g., *DOZI* and *ALBA1*, *-2*, and *-4*, which could potentially repress translation of transcripts important for late LS development and/or the subsequent transition to the ABS. Unfortunately, this possibility will be exceedingly difficult to test in the absence of robust global proteomic analysis of the early LS parasite. Nonetheless, our data, coupled with recent RNA-Seq and proteomic studies of the more accessible late LS, can provide a starting point to address this question (8, 32).

Previous work examining the transcriptional changes of axenically grown early LS P. vivax identified upregulation of calcium-related proteins (RACK1) and RNA-binding proteins (*ALBA1*, -*2* and -*4*) (33). We saw upregulation of the P. berghei orthologs of these genes, as well as hundreds of other genes, dramatically expanding the data set for genes upregulated at this stage (Fig. S5). For example, we found that *LYTB* is upregulated at 2 and 4 hpi, indicating isoprenoids may be important at this time. Although the FASII and *de novo* heme biosynthesis pathways have been genetically and chemically validated as essential to the late liver stages, less is known about isoprenoid biosynthesis during the early liver stages (34, 35). When intracellular sporozoites metamorphosize to replication-competent trophozoites, most organelles are expelled at the exclusion of the nucleus, mitochondrion, and apicoplast (10). Thus, it is plausible that the apicoplast serves an important metabolic role with isoprenoids in the liver stages of infection. Unfortunately, the use of isoprenoid biosynthesis inhibitors has yielded inconclusive results about its function during the LS (36, 37), emphasizing the need for future genetic studies to elucidate the role of isoprenoid biosynthesis throughout intrahepatic development. Thus, we anticipate our data will be useful to guide future reverse genetic and functional studies to investigate the role of *Plasmodium* genes with important early- and mid-LS functions.

10.1128/mBio.03234-19.5 FIG S5 Venn diagram illustrating the overlap of upregulated genes from our 4 hpi dataset and Roth et. al.’s P. vivax early-LS data (RPMI, 0 h versus RPMI 4 h, 37°C). P. berghei genes were translated into their respective syntenic P. vivax orthologs for this comparison (hypergeometric *P* value < 3.4E–06). We speculate that we do not see more overlap in upregulated genes because of (i) technical and experimental issues, such as the differences in coverage between the two datasets and the statistical cutoffs used to determine differential expression and (ii) biological differences, such as axenic culture versus intracellular developmental, and perhaps distinct temporal regulation of developmental programs between P. vivax and P. berghei. Download FIG S5, TIF file, 2.7 MB. Copyright © 2020 Toro-Moreno et al. 2020 Toro-Moreno et al. This content is distributed under the terms of the Creative Commons Attribution 4.0 International license.

Our understanding of *Plasmodium* LS biology still lags behind that of other parasite life cycle stages, hindering the development of much-needed prophylactic measures to combat malaria. Our work represents a window into the previously undescribed transcriptome of the early LS upon host cell infection and offers a comprehensive view of the *Plasmodium* LS. Future studies expanding on our analysis and validating time-specific LS genes will further advance our molecular understanding of this critical step in the *Plasmodium* life cycle.

## MATERIALS AND METHODS

### Parasites.

Sporozoites were freshly harvested prior to experiments from dissected salivary glands of Anopheles stephensi mosquitoes infected with P. berghei ANKA stably expressing a GFP purchased from the New York University Langone Medical Center Insectary.

### Cell culture.

HepG2 were purchased from ATCC and HuH7 cells were kindly provided by Peter Sorger (Harvard Medical School). Hepatocytes used for P. berghei infections were maintained in Dulbecco modified Eagle medium with l-glutamine (Gibco) supplemented with 10% (vol/vol) heat-inactivated fetal bovine serum (Sigma-Aldrich) and 1% antibiotic-antimycotic (Thermo Fisher Scientific) in a standard tissue culture incubator (37°C, 5% CO_2_).

### Sample collection for RNA-Seq.

Infected hepatoma cells were collected as previously described (38). Briefly, T25 flasks were seeded with 3 × 10^5^ HepG2 or 8 × 10^4^ HuH7 cells. About 24 h after seeding, the cells were infected with 1 × 10^5^ GFP-expressing P. berghei-ANKA sporozoites. Infected cells and uninfected controls were sorted directly into RNA lysis buffer (Clontech) using a BD FACSAria II cell sorter (BD Biosciences) at the Duke Human Vaccine Institute. Sytox Blue was used as a live/dead cell indicator (Thermo Fisher Scientific). Infected cells were collected by sorting of the GFP and gated compared to uninfected hepatoma cells. RNA was extracted using SMART-seq v4 Ultra Low Input RNA kit for sequencing (Clontech), and libraries were prepared at the Duke Next Generation Sequencing Core Facility and sequenced on the Illumina HiSeq 4000 as 50-bp single-end reads. Four or five samples were pooled on each flow cell lane.

### RNA-Seq and differential expression analysis.

RNA-Seq data were processed using a TrimGalore toolkit (39), which employs Cutadapt (40) to trim low-quality bases and Illumina sequencing adapters from the 3′ end of the reads. Only reads that were 20 nucleotides or longer after trimming were kept for further analysis. Reads were mapped to a combination of the GRCh37v75 (41) version of the human genome and the PbANKAv3 of the P. berghei genome using the STAR RNA-Seq alignment tool (42). Reads were kept for subsequent analysis if they mapped to a single genomic location. All samples mapping >1 million reads to the P. berghei genome were used for a preliminary analysis. Gene counts were compiled using the HTSeq tool (43). Only P. berghei genes that had at least 10 reads in any given library were used in subsequent analysis. Normalization and differential expression were carried out using the DESeq2 (44) Bioconductor (45) package with the R statistical programming environment (46). The false discovery rate (FDR) was calculated to control for multiple hypothesis testing. When calculating the differential expression between genes at each time point relative to the control, the cell type and sequencing batch were included as cofactors in the model.

Spearman correlations between published P. berghei RNA-Seq data sets and our own were calculated and plotted using the *cor* function in the *stats* R package.

### Clustering analysis.

To determine the different patterns of gene expression across all groups of samples, we first identified genes that showed differential expression in at least one of the comparisons performed (FDR ≤ 5%). The genes were the clustered across all samples by a correlation distance using complete linkage after z-score transformation. The *NbClust* (47) package was used to separate the gene expression across all samples into distinct clusters.

*De novo* motif discovery was performed using DREME from the MEME suite (23). For each cluster, the input data set was the upstream 1,000-kb region of each gene within that cluster, and the negative set was the upstream region of genes that were not in that cluster. The analysis was run in discriminative mode, scanning the given strand only, with the predicted motif size of 4 to 10 bp and a cutoff E value of 0.05. The top most enriched motif for each cluster was then analyzed with TOMTOM (48) to compare to previously *in silico*-discovered motifs (49).

The correlation matrix was generated in Prism by calculating the Pearson correlation between each AP2 transcription factor and the average fold change expression for all the genes in each cluster.

### Gene ontology.

GO analyses for each cluster were performed using the GO enrichment tool for Biological Processes in PlasmoDB (50) with a cutoff of *P* < 0.01. The number of genes from the cluster in each of the representative top-scoring GO terms (i.e., the lowest *P* values) were plotted.
