# Supplementary figures and images for: RNA-Seq Analysis Illuminates the Early Stages of Plasmodium Liver Infection
Source: mBio. 2020 Feb 4;11(1):e03234-19. doi: 10.1128/mBio.03234-19 (PMC7002348; doi:10.1128/mBio.03234-19)

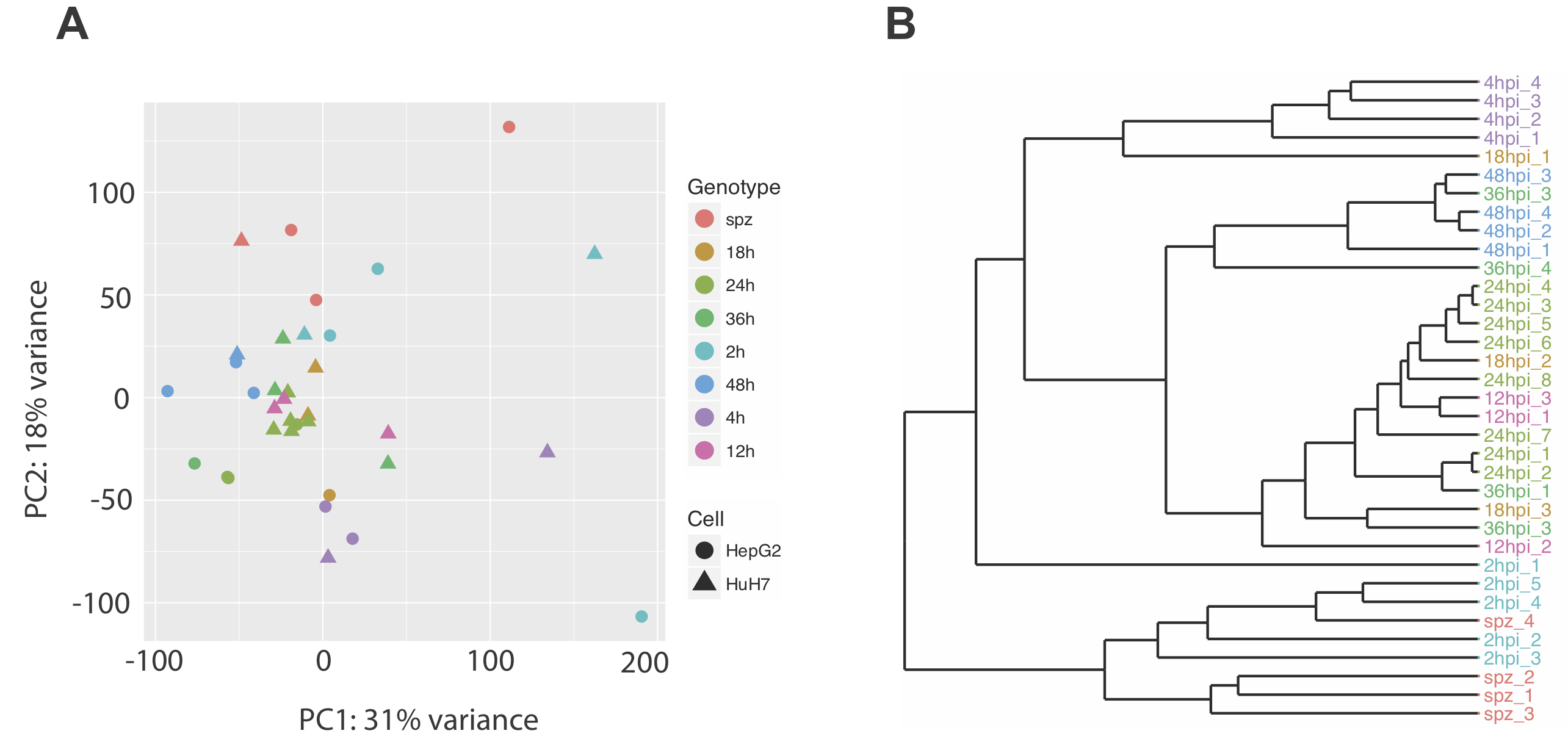

Supplement: FIG S1 [file mBio.03234-19-sf001.tif]

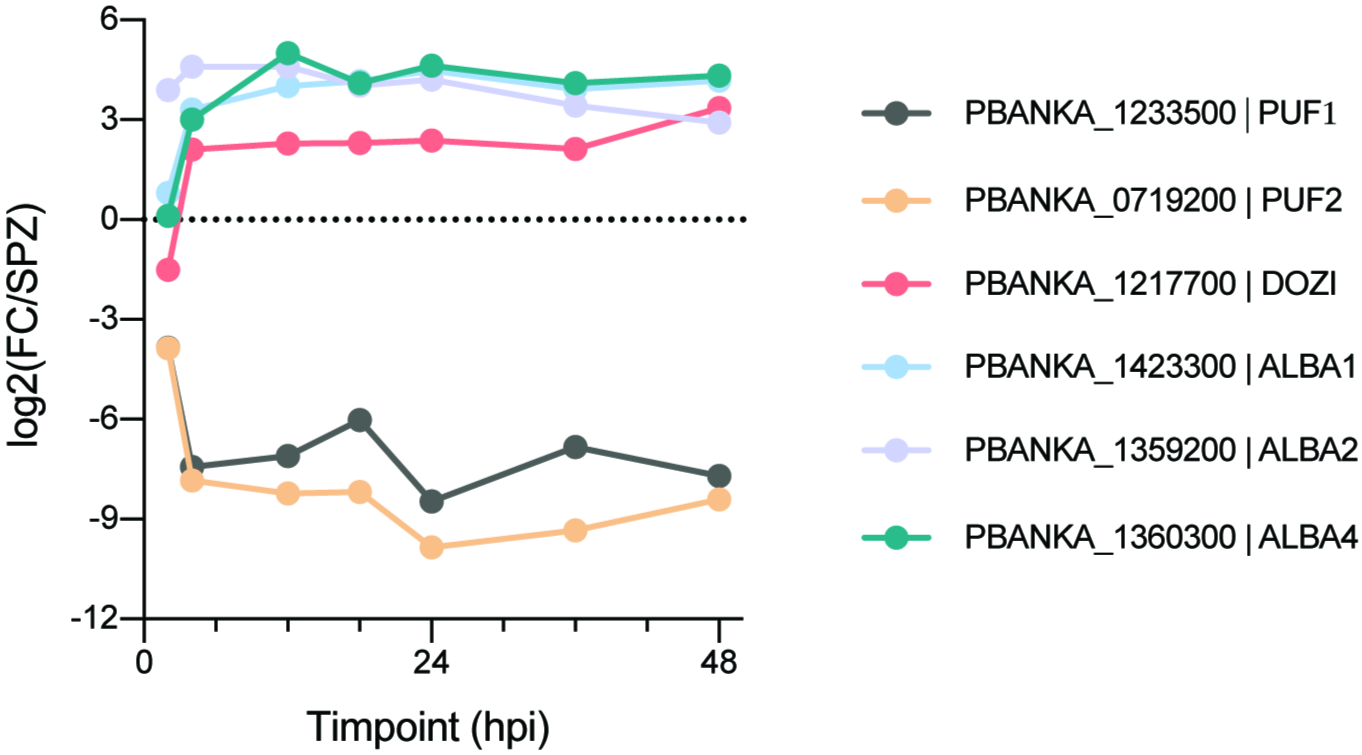

Supplement: FIG S2 [file mBio.03234-19-sf002.tif]

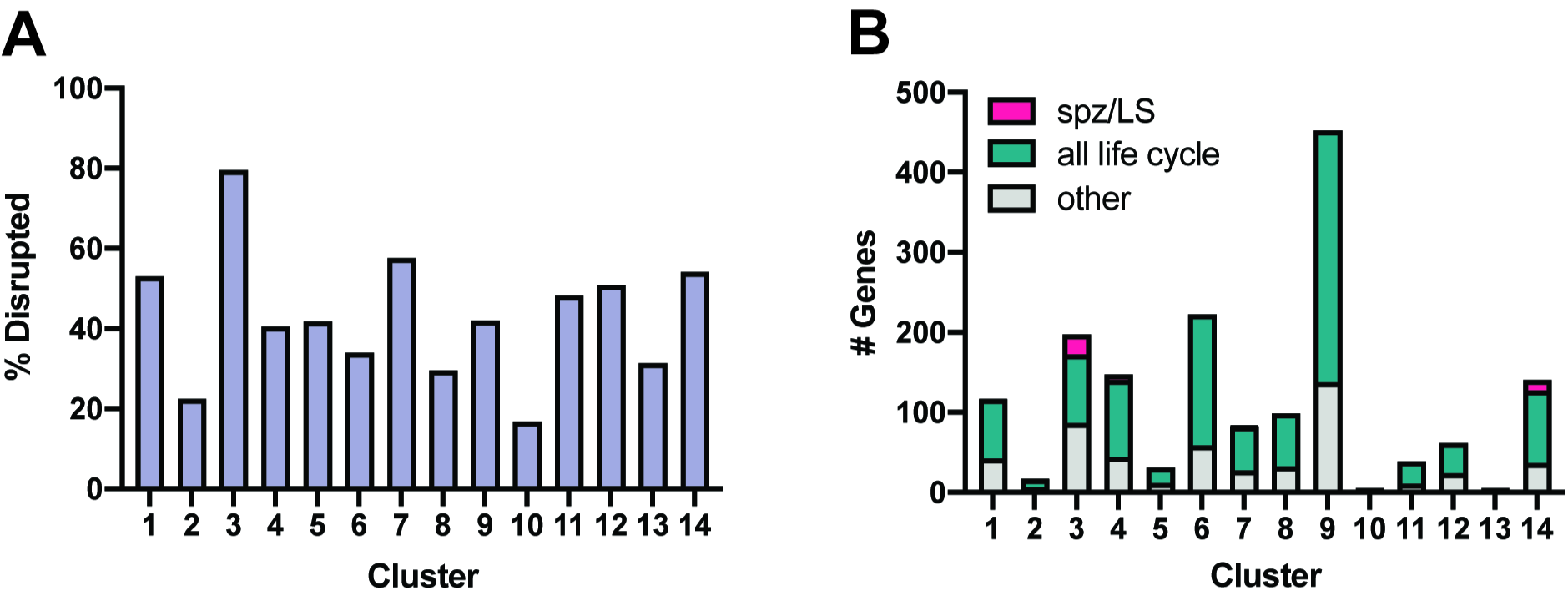

Supplement: FIG S3 [file mBio.03234-19-sf003.tif]

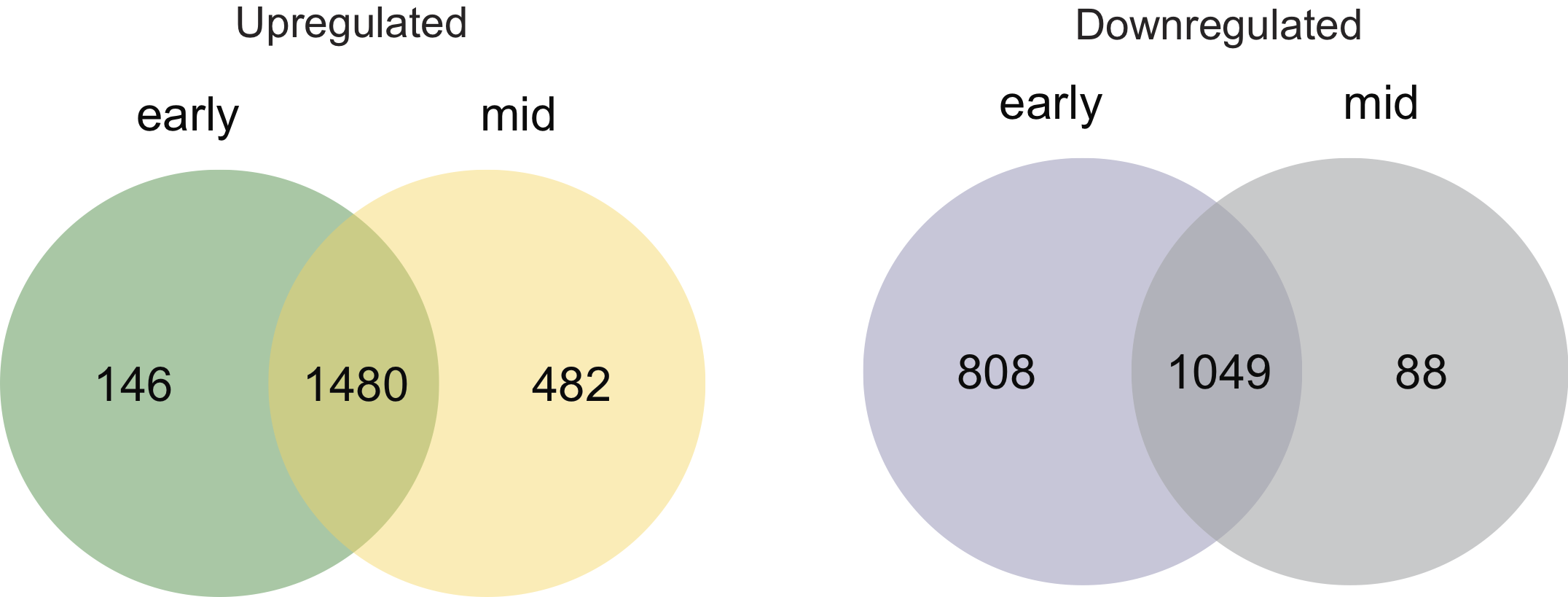

Supplement: FIG S4 [file mBio.03234-19-sf004.tif]

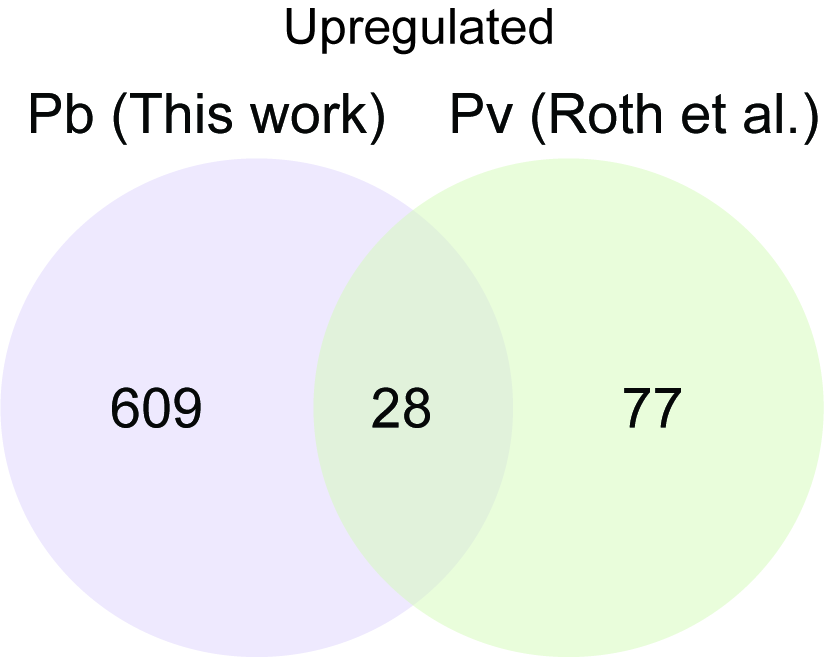

Supplement: FIG S5 [file mBio.03234-19-sf005.tif]
