# Supplementary material for: RNA-Seq Analysis Illuminates the Early Stages of Plasmodium Liver Infection
Source: mBio. 2020 Feb 4;11(1):e03234-19. doi: 10.1128/mBio.03234-19 (PMC7002348; doi:10.1128/mBio.03234-19)
Supplement: TABLE S1 [file mBio.03234-19-st001.docx]

**Table S1.** Datasets used in Spearman correlation analysis (Figure 2).

| **Source (ref)** | **Parasite Species** | **Samples** | **Host cell** |
| --- | --- | --- | --- |
| Zanghi (54) | *P. falciparum* | SgSpz, oocyst, ring |  |
| Gural (53) | *P. vivax* | hypnozoite (Hz), mixed LS | Primary human hepatocytes |
| Voorberg (55) | *P. cynomolgi* | LS (schizont), hypnozoite (Hz) | *M. mulatta* or *M. fascicularis* hepatocytes |
| Cubi (7) | *P. cynomolgi* | Hypnozoite (Hz), SgSpz, BS | *M. fascicularis* hepatocytes |
| Caldelari (8) | *P. berghei* | SgSpz, LS_24, LS_48, LS_54, LS_60 | HeLa |
| LaMonte (26) | *P. berghei* | SgSpz, LS_24, LS_48 | HuH7, HC04, HepG2 |
| Lindner (32) | *P. falciparum,*  *P. yoelii* | SgSpz, OoSpz |  |
| Roth (34) | *P. vivax* | SgSpz(0h), LS_4h |  |
| This work | *P. berghei* | SgSpz, LS_2h, LS_4h, LS_12h _LS_18h, LS_24h, LS_36h, LS_48h | HuH7 and HepG2 |
