# Supplementary material for: RNA-Seq Analysis Illuminates the Early Stages of Plasmodium Liver Infection
Source: mBio. 2020 Feb 4;11(1):e03234-19. doi: 10.1128/mBio.03234-19 (PMC7002348; doi:10.1128/mBio.03234-19)
Supplement: TABLE S2 [file mBio.03234-19-st002.docx]

**Table S2.** Top most upregulated genes at 4 hours post-infection.

| **Gene ID** | **Product Description** | **Gene Name** |
| --- | --- | --- |
| PBANKA_0201500 | Plasmodium exported protein, unknown function | N/A |
| PBANKA_0208700 | 4-hydroxy-3-methylbut-2-enyl diphosphate reductase, putative | LytB |
| PBANKA_0214600 | Plasmodium exported protein, unknown function | N/A |
| PBANKA_0315420 | Plasmodium RNA of unknown function RUF2 | N/A |
| PBANKA_0405400 | 40S ribosomal protein S12, putative | RPS12 |
| PBANKA_0405500 | 60S ribosomal protein L7, putative | N/A |
| PBANKA_0418500 | Plasmodium exported protein, unknown function | N/A |
| PBANKA_0517100 | conserved protein, unknown function | N/A |
| PBANKA_0602700 | nucleosome assembly protein, putative | NAPL |
| PBANKA_0619700 | rhoptry-associated leucine zipper-like protein 1, putative | RALP1 |
| PBANKA_0623200 | lysophospholipase, putative | N/A |
| PBANKA_0623500 | fam-a protein | N/A |
| PBANKA_0700500 | fam-a protein | N/A |
| PBANKA_0807100 | stomatin-like protein | STOML |
| PBANKA_0942100 | metabolite/drug transporter, putative | N/A |
| PBANKA_1011900 | H/ACA ribonucleoprotein complex subunit 3, putative | NOP10 |
| PBANKA_1025200 | H/ACA ribonucleoprotein complex subunit 4, putative | CBF5 |
| PBANKA_1030700 | conserved Plasmodium protein, unknown function | N/A |
| PBANKA_1101100 | Plasmodium exported protein, unknown function | N/A |
| PBANKA_1120800 | signal recognition particle subunit SRP68, putative | SRP68 |
| PBANKA_1127700 | nicotinate phosphoribosyl transferase, putative | NAPRT |
| PBANKA_1302241 | tRNA Selenocysteine | N/A |
| PBANKA_1326400 | glyceraldehyde-3-phosphate dehydrogenase | GAPDH |
| PBANKA_1347800 | 20 kDa chaperonin, putative | CPN20 |
| PBANKA_1365680 | fam-c protein | N/A |
| PBANKA_1407600 | 60S ribosomal protein L24, putative | N/A |
| PBANKA_1437100 | conserved Plasmodium protein, unknown function | N/A |
| PBANKA_1441700 | parasitophorous vacuolar protein 2 | PV2 |
