# Supplementary material for: RNA-Seq Analysis Illuminates the Early Stages of Plasmodium Liver Infection
Source: mBio. 2020 Feb 4;11(1):e03234-19. doi: 10.1128/mBio.03234-19 (PMC7002348; doi:10.1128/mBio.03234-19)
Supplement: TABLE S3 [file mBio.03234-19-st003.docx]

**Table S3.** Top most upregulated genes at 12 hours post-infection.

| **Gene ID** | **Product Description** | **Gene Name** |
| --- | --- | --- |
| PBANKA_0108900 | peptidyl-tRNA hydrolase PTRHD1, putative | N/A |
| PBANKA_0201000 | fam-b protein | N/A |
| PBANKA_0201500 | Plasmodium exported protein, unknown function | N/A |
| PBANKA_0211500 | proteasome subunit alpha type-5, putative | N/A |
| PBANKA_0315900 | pseudouridine synthase, putative | N/A |
| PBANKA_0404200 | ubiquitin-conjugating enzyme E2, putative | N/A |
| PBANKA_0406500 | T-complex protein 1 subunit eta, putative | CCT7 |
| PBANKA_0511900 | 60S ribosomal protein L3, putative | RPL3 |
| PBANKA_0517200 | haloacid dehalogenase-like hydrolase, putative | HAD1 |
| PBANKA_0619100 | 40S ribosomal protein S5, putative | N/A |
| PBANKA_0619300 | conserved Plasmodium protein, unknown function | N/A |
| PBANKA_0623200 | lysophospholipase, putative | N/A |
| PBANKA_0623500 | fam-a protein | N/A |
| PBANKA_0700500 | fam-a protein | N/A |
| PBANKA_0922800 | small nuclear ribonucleoprotein Sm D1, putative | SNRPD1 |
| PBANKA_1011900 | H/ACA ribonucleoprotein complex subunit 3, putative | NOP10 |
| PBANKA_1101100 | Plasmodium exported protein, unknown function | N/A |
| PBANKA_1101200 | Plasmodium exported protein, unknown function | N/A |
| PBANKA_1120200 | pyridoxine biosynthesis protein PDX1, putative | PDX1 |
| PBANKA_1301400 | proteasome subunit alpha type-1, putative | N/A |
| PBANKA_1302241 | tRNA Selenocysteine | N/A |
| PBANKA_1305100 | 60S ribosomal protein L1, putative | N/A |
| PBANKA_1326400 | glyceraldehyde-3-phosphate dehydrogenase | GAPDH |
| PBANKA_1360300 | DNA/RNA-binding protein Alba 4, putative | ALBA4 |
| PBANKA_1365680 | fam-c protein | N/A |
| PBANKA_1403000 | small heat shock protein, putative | N/A |
| PBANKA_1407600 | 60S ribosomal protein L24, putative | N/A |
| PBANKA_1446200 | mitosis protein dim1, putative | N/A |
